# Results of the Vulnerable Populations Tuberculosis Screening Project 2025 in the Republic of Korea

**DOI:** 10.56786/PHWR.2026.19.38.3

**Published:** 2026-09-01

**Authors:** Younghwa Kim, Heeae Kim, Yujin Kim, Seungeun Lee

**Affiliations:** Division of Tuberculosis Policy, Department of Infectious Disease Policy, Korea Disease Control and Prevention Agency Cheongju Korea

**Keywords:** Tuberculosis, Older adults, Homeless, Chest X-ray, Sputum test

## Abstract

**Objectives:**

This study aimed to analyze and report the results of the 2025 mobile tuberculosis (TB) screening program targeting vulnerable populations and to suggest directions for improving TB prevention and control policies for these groups.

**Methods:**

Screening followed a three-step process: a pre-screening questionnaire, chest X-ray examination, and sputum testing. Sputum testing was administered to individuals reporting TB-like symptoms or exhibiting suspicious findings on chest X-rays, including inactive TB lesions.

**Results:**

A total of 167,636 older adults participated in the screening program, yielding 119 identified TB cases. Among homeless individuals and jjokbang residents, 15,178 individuals participated, and 18 patients with TB were identified. In the older adult group, higher TB detection rates were observed among males, older age groups, individuals with symptoms suggestive of TB, and those with suspicious chest X-ray findings (p<0.001). Among homeless individuals and jjokbang residents, significant differences in detection rates were associated with poor living environments and suspicious chest X-ray findings (p<0.05). Notably, 81.0% of patients identified through the screening program reported having no cough lasting two weeks or longer.

**Conclusions:**

The mobile TB screening program is an effective policy tool for the early detection of TB among vulnerable populations and for linking patients to appropriate treatment. These findings provide empirical evidence for identifying high-risk groups for future screening initiatives. Expanding follow-up screening and maintaining continuous treatment support are necessary to maximize the effectiveness of the program.

**Key messages**

**① What is known previously?**

Although the overall tuberculosis (TB) incidence rate per 100,000 population has been decreasing, the proportion of older adults among all TB cases has steadily increased.

**② What new information is presented?**

In 2025, screening of 167,636 older adults identified 119 TB cases, while screening of 15,178 homeless individuals and jjokbang residents detected 18 TB cases. Notably, 81.0% of identified patients reported no cough lasting two weeks or longer yet were confirmed positive through sputum testing.

**③ What are implications?**

These findings demonstrate that the mobile TB screening program is an effective strategy for early detection and linkage to treatment among vulnerable populations with limited access to healthcare.

## Introduction

The Republic of Korea (ROK) is a country with a high tuberculosis (TB) incidence rate among Organisation for Economic Co-operation and Development countries, and despite a continuous declining trend, it remains a major infectious disease that still requires management [1]. As of 2025, the number of patients with TB was 17,070 (33.5 cases per 100,000 population), representing an average annual decrease of 7.5% since 50,491 in 2011 and maintaining a downward trend for 14 consecutive years; however, the declining trend in patients has recently shown signs of slowing. In particular, this slowing of the decline is prominent among older adults aged 65 and older. Despite the decrease in the incidence rate per 100,000 population due to the impact of various TB policies, the proportion of older adults among all patients with TB is continuously increasing from 51.3% in 2021 to 62.5% in 2025 due to the influence of ROK’s population aging, showing a shift in the disease structure centered on older adults. In addition, the TB burden among vulnerable populations also remains at a high level. The TB incidence rate among Medical Aid beneficiaries was 128.9 cases per 100,000 population, which was approximately 4.5 times higher than that of National Health Insurance enrollees (28.9 cases) [2]. This finding might be attributed to a combination of socioeconomic factors, including a lack of access to healthcare, nutritional status, and housing environment [3].

TB is a chronic infectious disease caused by *Mycobacterium tuberculosis* that is transmitted through airborne transmission; it primarily affects the lungs but can also occur in systemic organs [1]. In particular, the risk of occurrence is known to be high among older adults with weakened immunity and vulnerable populations [3]. If early detection and appropriate treatment are not provided, it can lead to community transmission, requiring continuous management [4].

Accordingly, the Korea Disease Control and Prevention Agency is actively promoting TB prevention and management policies targeting vulnerable populations. Notably, through the annual “Outreach TB Screening Program” since 2020, it provides screening opportunities to vulnerable populations with low healthcare access due to physical and socioeconomic reasons, and aims to block community transmission by strengthening early case detection and linkage to treatment. This study aims to analyze and report the results of the 2025 outreach TB screening program for vulnerable populations, including older adults, homeless individuals, and residents of jjokbang (single-room occupancy units), and based on these findings, suggest directions for improving TB prevention and management policies for vulnerable populations.

## Methods

Data from 182,814 of the 185,763 participants in the 2025 outreach TB screening program conducted from January to December 2025 were analyzed. The target population of this program included individuals assessed as long-term care grades 3, 4, and 5, aged 65 or older, Medical Aid beneficiaries, homeless individuals and jjokbang residents, and undocumented migrants (irregular/unauthorized foreign residents). However, for undocumented migrants, the screening was conducted after minimizing personal information and the pre-screening questionnaire due to concerns over identity exposure, and the 2,949 individuals in this category were excluded from the final analysis. To identify the general characteristics and screening results of the participants, they were presented as frequencies and percentages, and significant differences in the TB case detection rate according to subject characteristics were analyzed using the chi-square test (χ^2^ test).

In this program, the contracted screening institution (Korean National Tuberculosis Association) directly visited the institutions, facilities, and homes used by the target population to improve the convenience and accessibility of TB screening. The screening methods comprised hub-based screening using mobile screening vans and visiting screening using portable chest X-ray equipment.

The screening procedure consisted of a pre-screening questionnaire, a chest X-ray examination, and a sputum test, in that order. Through the pre-screening questionnaire, personal information, suspected TB symptoms (such as coughing for 2 weeks or longer), and underlying diseases were investigated. In the case of the chest X-ray examination, the doctor’s interpretation results were provided on the same day through immediate post-imaging artificial intelligence interpretation and a remote interpretation system. For sputum tests, sputum was collected from individuals who either responded on the pre-screening questionnaire that they had suspected TB symptoms or had findings suggestive of TB (including inactive TB) on a chest X-ray examination, to perform a sputum smear test, sputum culture test, and nucleic acid amplification test for *M. tuberculosis* (Tuberculosis Polymerase Chain Reaction). If any one of these cases was confirmed positive, they were classified as TB cases.

Among the homeless individuals and jjokbang residents who participated in the screening program, those confirmed as patients with TB could apply for remote medication management. For those applicants, a coordinator from the contracted screening institution managed their medication via remote video or telephonic conversation. When a monthly medication adherence rate of 80% or higher was maintained, groceries and daily necessities (KRW 100,000 per month) were provided for up to 6 months to promote treatment continuity and improve success. In addition, various treatment incentives, such as temporary housing support costs, resident registration reinstatement fees, and private ambulance service fees, were provided. The relevant incentive was provided after an individual was confirmed as a patient with TB through the screening program applied to a public health center and underwent a verification procedure by the contracted screening institution.

## Results

### 1. General Characteristics of Participants and Detected Patients in the Older Adults Screening Program

In 2025, there were a total of 167,636 participants in the outreach TB screening program for older adults: 72.4% (121,384) were female, 66.4% (111,294) were aged 80 or older, 59.5% (99,674) were individuals with limited mobility, 48.9% (81,943) lived alone, 73.2% (122,630) had underlying medical conditions, 3.2% (5,351) had a cough lasting 2 weeks or longer (symptomatic individuals), and 8.9% (14,865) had chest X-ray findings suggestive of TB.

The TB case detection rate was 71.0 cases (per 100,000 screenings), showing statistically significant differences in males, older age (80 years and older), individuals with TB symptoms, and individuals with findings suggestive of TB on chest X-ray interpretation (p<0.001) (Table 1).

### 2. General Characteristics of the Participants in the Screening Program and Detected Patients among Homeless Individuals and Jjokbang Residents

In 2025, a total of 15,178 individuals participated in the outreach TB screening program for homeless individuals and jjokbang residents, consisting of 77.6% males (11,776 individuals), 66.6% aged 60 or older (10,108 individuals), 44.2% sheltered homeless individuals (6,704 individuals), 50.8% with underlying diseases (7,707 individuals), 1.4% symptomatic individuals (215 individuals), and 11.8% with chest X-ray interpretation findings suggestive of TB (1,794 individuals).

The TB case detection rate was 118.6 cases (per 100,000 screenings), and those with poor housing conditions (participants who responded as “street homeless” or “jjokbang residents” in the screening target classification during the pre-screening questionnaire) and those with chest X-ray interpretation findings suggestive of TB showed statistically significant differences (p<0.05) (Table 2). However, caution should be exercised while generalizing the results due to the small number of patients detected through the screening program.

### 3. Status of the Detected Confirmed TB Cases by Symptoms and Findings

Among the 182,814 participants of the screening program in 2025, 137 were confirmed to have TB, of which 81.0% (111 individuals) had asymptomatic TB, responding in the pre-screening questionnaire that they had no cough symptoms for 2 weeks or longer. Among all patients, 75.2% (103 individuals) were interpreted as having inactive TB based on chest X-ray interpretation, of which 87.4% (90 individuals) were ultimately confirmed to have asymptomatic TB (Table 3).

## Discussion

This study aimed to analyze the operational results of the outreach TB screening program conducted for vulnerable populations in 2025 to derive the program’s effectiveness and future policy directions.

Of the total 185,763 individuals who participated in the 2025 outreach TB screening program, 182,814 were analyzed, excluding undocumented migrants with unclear pre-screening questionnaires. Not a single TB case was detected among the undocumented migrants who participated in the screening.

While screening for older adults, a total of 167,636 individuals participated and 119 patients with TB (71.0 cases per 100,000 screenings) were detected, which was slightly lower (0.9 times) but similar to the incidence rate (79.5 cases per 100,000 population) among those aged 65 or older (8,486 individuals) among patients with pulmonary TB (excluding extrapulmonary TB) reported in 2025 [2]. While screening for homeless individuals and jjokbang residents, a total of 15,178 individuals participated, and 18 TB cases (118.6 cases per 100,000 screenings) were detected. This incidence was slightly higher (1.1 times) but comparable to the incidence rate (104.8 cases per 100,000 population) among Medical Aid beneficiaries (1,635 individuals) among patients with pulmonary TB (excluding extrapulmonary TB) reported in 2025 [2]. This finding demonstrated that active screening through an outreach approach is as effective a strategy for early case detection as the existing medical institution–centered passive case detection system.

While screening for older adults, the TB case detection rate was higher among males, older age groups, those with suspected TB symptoms, and those with chest X-ray findings suggestive of TB (p<0.001). While screening for homeless individuals and jjokbang residents, significant differences were observed in the TB case detection rate for those with poor housing environments and those with chest X-ray findings suggestive of TB (p<0.05). This finding indicates that the probability of TB occurrence is high in groups with complex risk factors such as advanced age, housing vulnerability, and health vulnerability [3], which implies the need to establish priorities reflecting these characteristics when selecting target subjects in the future.

In particular, 81.0% (111 individuals) of the 137 patients detected through the screening program responded that they did not have cough symptoms lasting for 2 weeks or longer, confirming the importance of early detection for patients with asymptomatic TB. Symptom-based screening alone makes it difficult to detect a significant number of patients early, and if the disease progresses in an asymptomatic state, it may lead to diagnostic delays and community transmission [5]. Therefore, it is necessary to maintain and strengthen a regular and active screening system targeting high-risk groups.

In addition, this program covered a wider scope than the existing national health screening system by expanding sputum tests to include individuals who showed manifestations of inactive TB on chest X-ray examinations. While the current National General Health Checkup program classifies inactive TB within the normal range and excludes it from additional testing, this program was able to detect additional patients with TB by conducting confirmatory diagnostic tests (sputum tests), including those with suspected TB findings. This result demonstrates the need to operate screening criteria for high-risk groups more actively, and it can be used as evidence to refer to when improving the national health screening system in the future.

The 2026 outreach TB screening program is being implemented with further strengthened target selection and follow-up management systems. The scope of application for elderly screening participants has been expanded to those assessed as long-term care grades 1–5, and includes older adults who have difficulty using medical institutions and socioeconomically vulnerable older adults selected according to the characteristics of local governments. If there were findings suggestive of TB on the initial chest X-ray, even if the sputum test was negative, early case detection will be strengthened by emphasizing the implementation of follow-up screening (chest X-ray examination) within 6 months (for those who responded that they had cough symptoms for 2 weeks or longer when completing the pre-screening questionnaire, or had findings suggestive of TB [including inactive TB] on chest X-ray interpretation and thus underwent sputum testing but were ultimately confirmed as “negative”). For patients with TB among homeless individuals and jjokbang residents, we plan to continue operating treatment support policies such as temporary housing support costs and medication adherence management to increase treatment adherence and prevent recurrence and transmission due to treatment interruption. Through this screening-treatment-management system, we aim to further increase the effectiveness of TB management for vulnerable populations.

The outreach TB screening program is an effective joint national-local government policy tool for early detection and linkage to care of patients with TB within vulnerable populations. It can be utilized as baseline data for selecting screening targets centered on high-risk groups in the future, and there is a need to further enhance the effectiveness of the program by expanding follow-up screenings and continuing treatment support. Through this approach, we can prevent community transmission in advance and contribute to reducing the national TB incidence rate.

## Figures and Tables

**Table 1:** General characteristics of participants in the tuberculosis screening program and confirmed tuberculosis cases among older adults

Variable	Participant		TB patient		
	Case (%)		Case (%)	Detection rate	p-value
Total	167,636 (100.0)		119 (100.0)	[71.0]	
Sex					<0.001
Male	46,252 (27.6)		55 (46.2)	[118.9]	
Female	121,384 (72.4)		64 (53.8)	[52.7]	
Age group					<0.001
65–69	9,163 (5.5)		7 (5.9)	[76.4]	
70–74	17,838 (10.6)		3 (2.5)	[16.8]	
75–79	29,341 (17.5)		12 (10.1)	[40.9]	
≥80	111,294 (66.4)		97 (81.5)	[87.2]	
Physical activity					0.675
Normal	67,962 (40.5)		46 (38.7)	[67.7]	
Mobility difficulties	99,674 (59.5)		73 (61.3)	[73.2]	
Living alone					0.879
Yes	81,943 (48.9)		59 (49.6)	[72.0]	
No	85,693 (51.1)		60 (50.4)	[70.0]	
Underlying disease					0.465
Yes	122,630 (73.2)		87 (73.1)	[70.9]	
No	42,954 (25.6)		32 (26.9)	[74.5]	
Unknown	2,052 (1.2)		0 (0.0)	[0.0]	
TB symptoms (cough lasting ≥2 weeks)					<0.001
Yes	5,351 (3.2)		26 (21.8)	[485.9]	
No	162,285 (96.8)		93 (78.2)	[57.3]	
Chest X-ray					<0.001
Normal	125,703 (75.0)		3 (2.5)	[2.4]	
Need treatmenta)/observation requiredb)	866 (0.5)		20 (16.8)	[2,309.5]	
Inactive TBc)	13,999 (8.4)		88 (73.9)	[628.6]	
Other diseases suspected	27,068 (16.1)		8 (6.7)	[29.6]	

Unit: case (%) or [case/10^5^ cases]. Percentages may not sum to 100% due to rounding. TB=tuberculosis. ^a)^“Active pulmonary tuberculosis” or “exudative pleural effusion” presumed to be tuberculosis, indicating the need for tuberculosis treatment and sputum examintaion for confirmation. ^b)^Any suspicion of “active tuberculosis” or “suspected tuberculosis,” requiring a final diagnosis by a physician based on additional tuberculosis testing, including sputum examination, and the patient’s clinical findings at public health centers and medical institutions. ^c)^Pulmonary tuberculosis that developed in the past but has healed, with traces of fibrotic changes.

**Table 2:** General characteristics of participants in the tuberculosis screening program and confirmed tuberculosis cases among homeless individuals and jjokbang residents

Variable	Participant		TB patient		
	Case (%)		Case (%)	Detection rate	p-value
Total	15,178 (100.0)		18 (100.0)	[118.6]	
Sex					0.086
Male	11,776 (77.6)		17 (94.4)	[144.4]	
Female	3,402 (22.4)		1 (5.6)	[29.4]	
Age group					0.827
≤39	530 (3.5)		0 (0.0)	[0.0]	
40–49	1,178 (7.8)		1 (5.6)	[84.9]	
50–59	3,362 (22.2)		6 (33.3)	[178.5]	
60–69	5,158 (34.0)		7 (38.9)	[135.7]	
70–79	3,331 (21.9)		2 (11.1)	[60.0]	
≥80	1,619 (10.7)		2 (11.1)	[123.5]	
Participants screened					0.019
Sheltered homeless people	6,704 (44.2)		3 (16.7)	[44.7]	
Homeless people living on the streets	3,817 (25.1)		5 (27.8)	[131.0]	
Jjokbang residents	4,657 (30.7)		10 (55.6)	[214.7]	
Underlying disease					0.273
Yes	7,707 (50.8)		6 (33.3)	[77.9]	
No	7,292 (48.0)		12 (66.7)	[164.6]	
Unknown	179 (1.2)		0 (0.0)	[0.0]	
TB symptoms (cough lasting ≥2 weeks)					0.611
Yes	215 (1.4)		0 (0.0)	[0.0]	
No	14,963 (98.6)		18 (100.0)	[120.3]	
Chest X-ray					<0.001
Normal	12,072 (79.5)		0 (0.0)	[0.0]	
Need treatment/observation required	73 (0.5)		3 (16.7)	[4109.6]	
Inactive TB	1,721 (11.3)		15 (83.3)	[871.6]	
Other diseases suspected	1,312 (8.6)		0 (0.0)	[0.0]	

Unit: case (%) or [case/10^5^ cases]. Percentages may not sum to 100% due to rounding. TB=tuberculosis.

**Table 3:** Detection of confirmed tuberculosis cases according to symptom status and chest X-ray findings

Chest X-ray		TB patient	TB symptoms (cough lasting ≥2 weeks)	
			Symptomatic patients	Asymptomatic patients
Total	Total	137 (100.0)	26 (19.0)	111 (81.0)
	Normal	3 (2.2)	3 (100.0)	0 (0.0)
	Need treatment/observation required	23 (16.8)	2 (8.7)	21 (91.3)
	Inactive TB	103 (75.2)	13 (12.6)	90 (87.4)
	Other diseases suspected	8 (5.8)	8 (100.0)	0 (0.0)
Older adults	Subtotal	119 (100.0)	26 (21.8)	93 (78.2)
	Normal	3 (2.5)	3 (100.0)	0 (0.0)
	Need treatment/observation required	20 (16.8)	2 (10.0)	18 (90.0)
	Inactive TB	88 (73.9)	13 (14.8)	75 (85.2)
	Other diseases suspected	8 (6.7)	8 (100.0)	0 (0.0)
Homeless individuals and jjokbang residents	Subtotal	18 (100.0)	0 (0.0)	18 (100.0)
	Normal	0 (0.0)	0 (0.0)	0 (0.0)
	Need treatment/observation required	3 (16.7)	0 (0.0)	3 (100.0)
	Inactive TB	15 (83.3)	0 (0.0)	15 (100.0)
	Other diseases suspected	0 (0.0)	0 (0.0)	0 (0.0)

Unit: case (%). Percentages may not sum to 100% due to rounding. TB=tuberculosis.
