# Supplementary material for: Results of the Vulnerable Populations Tuberculosis Screening Project 2025 in the Republic of Korea
Source: Public Health Wkly Rep. 2026 Sep 1;19(38):1633–47. doi: 10.56786/PHWR.2026.19.38.3 (PMC13654658; doi:10.56786/PHWR.2026.19.38.3)
Supplement: Translation

# Translation: 2025년 취약계층 대상 결핵검진 사업 결과 분석

**Authors:** 영화 김, 희애 김, 유진 김, 승은 이

**Affiliations:** 질병관리청 감염병정책국 결핵정책과

**Keywords:** 결핵, 노인, 노숙인, 흉부X선 검사, 가래검사

## Abstract

**목적:**

질병관리청은 ‘찾아가는 결핵검진’ 사업을 통해 의료 접근성이 낮은 대상자에게 검진 기회를 제공하고, 환자 조기 발견 및 치료 연계를 강화하여 지역사회 전파 차단을 도모하고 있다. 본 연구에서는 2025년에 실시한 취약계층 대상 찾아가는 결핵검진 사업의 결과를 분석‧보고하고, 이를 바탕으로 취약계층 대상 결핵 예방관리 정책의 개선 방향을 제시하고자 한다.

**방법:**

본 사업은 결핵검진의 편의성과 접근성을 높이기 위해 대상자가 이용하는 시설 및 자택 등을 직접 방문하여 사전문진, 흉부X선 검사, 가래검사 순으로 진행하였다. 가래검사는 결핵 의심 증상이 있거나 흉부X선 검사에서 결핵 의심 소견(비활동성 결핵 포함)이 있는 경우 실시하였다.

**결과:**

노인 대상 검진에는 총 167,636명이 참여하여 119명(10만 건당 71.0명)의 결핵환자를 발견하였고, 노숙인 및 쪽방거주자 대상 검진에는 총 15,178명이 참여하여 18명(10만 건당 118.6명)의 결핵 환자를 발견하였다. 노인 대상 검진에서는 남자, 고연령, 결핵 의심 증상이 있는 경우, 흉부X선 결핵 의심 소견자에서 환자 발견율이 높게 나타났으며(p<0.001), 노숙인 및 쪽방거주자 대상에서는 주거 환경이 열악한 경우와 흉부X선 결핵 의심 소견자에서 유의한 차이를 보였다(p<0.05). 또한 검진사업을 통해 발견된 환자 중 81.0%가 2주 이상 기침 증상이 없었다고 응답하였다.

**결론:**

찾아가는 결핵검진 사업은 취약계층 내 결핵환자를 조기에 발견하고 치료로 연계하는 데 효과적인 정책 수단으로 향후 고위험군 중심의 검진 대상 선정에 근거 자료로 활용할 수 있고, 추적검진의 확대 및 치료 지원 지속 등을 통해 사업의 효과를 더욱 높일 필요가 있다.

**핵심요약**

**① 이전에 알려진 내용은?**

우리나라 인구 10만 명당 결핵발생률은 지속 감소하고 있으나, 인구 고령화의 영향으로 전체 결핵환자 중 고령층이 차지하는 비중은 2021년 51.3%에서 2025년 62.5%로 꾸준히 증가하고 있다.

**② 새로이 알게 된 내용은?**

2025년도 취약계층 대상 찾아가는 결핵검진 사업을 통해, 노인 총 167,636명이 참여하여 119명(10만 건당 71.0명), 노숙인 및 쪽방거주자 총 15,178명이 참여하여 18명(10만 건당 118.6명)의 결핵환자를 발견하였다. 발견된 환자 중 81.0%는 2주 이상 기침 증상이 없었다고 응답하였으나, 가래검사를 통해 결핵으로 확인되었다.

**③ 시사점은?**

찾아가는 결핵검진은 신체적ㆍ사회경제적 사유로 의료접근성이 낮은 취약계층을 찾아가서 검진 기회를 제공하여 결핵환자의 조기 발견 및 조기 치료로 연계하는 중요한 사업이다. 특히 발견한 환자 중 무증상 결핵이 많은 점에서, 결핵 고위험 취약계층에 대해서는 정기적인 결핵검진이 필요하다.

## 서 론

우리나라는 경제협력개발기구(Organisation for Economic Co-operation and Development) 국가 중 결핵 발생률이 높은 국가로, 지속적인 감소 추세에도 불구하고 여전히 관리가 필요한 주요 감염병으로 남아 있다[1]. 2025년 기준 결핵환자 수는 17,070명(인구 10만 명당 33.5명)으로, 2011년 50,491명 이후 연평균 7.5%씩 감소하여 14년 연속 감소세를 유지하고 있으나, 최근에는 환자 감소 추세가 둔화하는 양상이다. 특히 65세 이상 고령층에서 이러한 감소 둔화 현상이 두드러지는데, 다양한 결핵 정책의 영향으로 인구 10만 명당 발생률은 감소하고 있음에도 불구하고 우리나라 인구 고령화의 영향으로 전체 결핵환자 중 고령층이 차지하는 비중이 2021년 51.3%에서 2025년 62.5%로 지속 증가하는 고령층 중심의 질병 구조 변화를 보이고 있다. 또한, 취약계층에서의 결핵 부담 역시 여전히 높은 수준이다. 의료급여 수급권자의 결핵 발생률은 인구 10만 명당 128.9명으로, 건강보험 가입자(28.9명)에 비해 약 4.5배 높게 나타났다[2]. 이는 의료 접근성 부족, 영양 상태, 주거 환경 등 사회경제적 요인이 복합적으로 작용한 결과로 해석된다[3].

결핵은 결핵균(*Mycobacterium tuberculosis*)에 의해 발생하는 만성 감염병으로, 공기 전파를 통해 감염되며 주로 폐를 침범하지만 전신 장기에도 발생할 수 있다[1]. 특히 면역력이 저하된 고령층과 취약계층에서 발생 위험이 높은 것으로 알려져 있다[3]. 조기 발견과 적절한 치료가 이루어지지 않을 경우 지역사회 내 전파로 이어질 수 있어 지속적인 관리가 필요하다[4].

이에 질병관리청은 취약계층을 대상으로 한 결핵 예방 및 관리 정책을 적극적으로 추진하고 있는데, 대표적으로 2020년부터 매년 ‘찾아가는 결핵검진’ 사업을 통해 신체적·사회경제적 이유로 의료접근성이 낮은 취약계층에게 검진 기회를 제공하고, 환자 조기 발견 및 치료 연계를 강화하여 지역사회 전파 차단을 도모하고 있다. 본 연구에서는 2025년에 수행한 노인·노숙인 및 쪽방거주자 등 취약계층 대상 찾아가는 결핵검진 사업의 결과를 분석·보고하고, 이를 바탕으로 취약계층 대상 결핵 예방관리 정책의 개선 방향을 제시하고자 한다.

## 방 법

2025년 1월부터 12월까지 실시한 2025년 찾아가는 결핵검진 사업 참여자 185,763명 중 182,814명의 자료를 활용하여 분석하였다. 본 사업의 대상은 65세 이상 장기요양 3·4·5등급 판정자 및 의료급여 수급권자, 노숙인 및 쪽방거주자, 무자격체류자(불법 체류 외국인)이나, 무자격체류자의 경우 신분 노출 우려로 개인정보 및 사전문진을 최소화 후 수행하여 이에 해당하는 2,949명은 최종 분석 대상에서 제외하였다. 대상자의 일반적 특성과 검진 결과를 파악하기 위해 빈도와 백분율로 제시하였으며, 대상자 특성에 따른 결핵환자 발견율의 유의미한 차이는 카이제곱검정(χ^2^ test)을 이용하여 분석하였다.

본 사업은 결핵검진의 편의성과 접근성을 높이기 위해 대상자가 이용하는 기관·시설 및 자택 등으로 검진수탁기관(대한결핵협회)이 직접 방문하는 방식으로 수행하였다. 검진 방법은 이동검진 차량을 이용한 거점 검진과 휴대용 흉부X선 장비를 활용한 방문 검진으로 운영하였다.

검진 절차는 사전문진, 흉부X선 검사, 가래검사 순이었으며, 사전문진을 통해 인적 사항, 결핵 의심 증상(2주 이상 기침 등), 기저질환 등을 조사하였고, 흉부X선 검사 시 촬영 직후 인공지능(artificial intelligence) 판독 및 원격 판독 시스템을 통해 의사의 판독 결과를 당일 제공하였다. 가래검사는 사전문진에서 결핵 의심 증상이 있다고 응답하거나 흉부X선 검사에서 결핵 의심 소견(비활동성 결핵 포함)이 있는 자를 대상으로 가래를 채취하여 도말검사, 배양검사, 결핵균핵산증폭검사(Tuberculosis Polymerase Chain Reaction)를 시행하고, 그중 한 가지라도 양성으로 확인된 경우에는 결핵환자로 분류하였다.

검진사업에 참여한 노숙인 및 쪽방거주자 중 결핵환자로 확인된 경우 원격복약관리를 신청할 수 있으며, 신청자에 한해 검진수탁기관 코디네이터가 원격화상 또는 유선을 통해 복약을 관리하였다. 월 80% 이상 복약을 유지한 경우 식료품 및 생필품(월 10만 원)을 최대 6개월간 지원하여 치료 지속과 성공 향상을 도모하였다. 이 외에도 임시주거비, 주민등록 복원비, 사설 응급차량비 등 다양한 치료 인센티브를 제공하였다. 해당 인센티브는 검진사업을 통해 결핵환자로 확인된 대상자가 보건소에 신청하고, 검진수탁기관의 확인 절차를 거쳐 지원하였다.

## 결 과

### 1. 노인 검진사업 참여자 및 발견환자의 일반적 특성

2025년 노인 대상 찾아가는 결핵검진 사업 참여자는 총 167,636명으로 여자 72.4% (121,384명), 80세 이상 66.4% (111,294명), 거동 불편자 59.5% (99,674명), 혼자 거주하는 경우 48.9% (81,943명), 기저질환이 있는 경우 73.2% (122,630명), 2주 이상 기침 증상이 있는 경우(유증상자) 3.2% (5,351명), 흉부X선 판독 결과 결핵 의심 소견자인 경우가 8.9% (14,865명)였다.

결핵환자 발견율은 71.0명(검진 10만 건당)으로 남자, 고연령(80세 이상), 결핵 증상이 있는 경우, 흉부X선 판독 결과 결핵 의심 소견자인 경우에서 통계적으로 유의미한 차이를 보였다(p<0.001) (표 1).

### 2. 노숙인 및 쪽방거주자 검진사업 참여자 및 발견환자의 일반적 특성

2025년 노숙인 및 쪽방거주자 대상 찾아가는 결핵검진 사업 참여자는 총 15,178명으로 남자 77.6% (11,776명), 60세 이상 66.6% (10,108명), 시설노숙인 44.2% (6,704명), 기저질환이 있는 경우 50.8% (7,707명), 유증상자 1.4% (215명), 흉부X선 판독 결과 결핵 의심 소견자인 경우가 11.8% (1,794명)였다.

결핵환자 발견율은 118.6명(검진 10만 건당)으로 주거지가 열악한 경우^1)^, 흉부X선 판독 결과 결핵 의심 소견자가 통계적으로 유의미하였다(p<0.05) (표 2). 다만 검진사업을 통해 발견한 환자의 수가 적어서 일반화에 유의해야 한다.

### 3. 유증상-유소견에 따른 결핵 확진자 발견 현황

2025년 검진사업 참여자 182,814명 중 137명이 결핵으로 확진되었고, 그중 81.0% (111명)가 사전문진에서 2주 이상 기침 증상이 없다고 응답한 무증상 결핵이었다. 전체 환자 중 75.2% (103명)는 흉부X선 판독 결과 비활동성 결핵으로 판독된 경우였고, 그중 87.4% (90명)는 최종적으로 무증상 결핵으로 확인되었다(표 3).

## 논 의

본 연구는 2025년 취약계층을 대상으로 시행된 찾아가는 결핵검진 사업의 운영 결과를 분석하여 사업의 효과 및 향후 정책 방향을 도출하고자 하였다.

2025년도 찾아가는 결핵검진 사업에 참여한 총 185,763명 중 사전문진이 불명확한 무자격체류자를 제외한 182,814명에 대해 분석하였다. 검진에 참여한 무자격체류자 중에서는 결핵환자가 단 한 명도 발견되지 않았다.

노인 대상 검진에는 총 167,636명이 참여하여 119명(10만 건당 71.0명)의 결핵환자를 발견하였는데, 2025년에 신고된 폐결핵 환자(폐외결핵 제외) 중 65세 이상(8,486명)의 발생률(인구 10만 명당 79.5명)과 비교했을 때 0.9배로 약간 낮았으나 비슷한 수준이었다[2]. 노숙인 및 쪽방거주자 대상 검진에는 총 15,178명이 참여하였고, 18명(10만 건당 118.6명)의 결핵환자를 발견하였다. 이는 2025년 신고된 폐결핵 환자(폐외결핵 제외) 중 의료급여수급권자(1,635명)의 발생률(인구 10만 명당 104.8명)과 비교했을 때 1.1배로 약간 높았으나 비슷한 수준이었다[2]. 이는 기존 의료기관 중심의 수동적 환자 발견 체계뿐만 아니라 찾아가는 방식의 능동적 검진도 환자 조기 발견에 효과적인 전략임을 보여주었다.

노인 대상 검진에서는 남자, 고연령, 결핵 의심 증상이 있는 경우, 흉부X선 결핵 의심 소견자에서 환자 발견율이 높게 나타났으며(p<0.001), 노숙인 및 쪽방거주자 대상에서는 주거 환경이 열악한 경우와 흉부X선 결핵 의심 소견자에서 유의한 차이를 보였다(p<0.05). 이를 통해 고령, 주거 취약, 건강 취약 등 복합적인 위험요인을 가진 집단에서 결핵 발생 가능성이 높음을 알 수 있으며[3], 향후 대상자 선정 시 이러한 특성을 반영한 우선순위 설정이 필요함을 의미한다.

특히 검진사업을 통해 발견된 환자 137명 중 81.0% (111명)가 2주 이상 기침 증상이 없었다고 응답하여, 무증상 결핵환자에 대한 조기 발견 중요성을 확인하였다. 증상 기반의 선별 방식만으로는 상당수 환자를 조기에 발견하기 어려우며, 무증상 상태에서 질병이 진행될 경우 진단 지연 및 지역사회 전파로 이어질 가능성이 있다[5]. 따라서 고위험군을 대상으로 한 정기적이고 적극적인 검진 체계를 유지·강화할 필요가 있다.

또한 본 사업은 흉부X선 검사에서 비활동성 결핵 소견을 보인 대상자까지 가래검사를 확대 적용함으로써 기존 국가건강검진 체계보다 넓은 범위를 포괄하였다. 현재 국가 일반건강검진에서는 비활동성 결핵을 정상 범위로 분류하고 추가 검사 대상에서 제외하는 반면, 본 사업에서는 결핵 의심 소견자를 포함하여 확인진단검사(가래검사)를 시행하며 결핵환자를 추가로 발견할 수 있었다. 이는 고위험군을 대상으로 한 검진 기준을 보다 적극적으로 운영할 필요성을 보여주는 결과로 향후 국가건강검진 체계 개선 시 참고할 수 있는 근거로 활용될 수 있다.

2026년도 찾아가는 결핵검진 사업은 대상자 선정과 사후관리 체계를 보다 강화하여 수행하고 있다. 노인 검진 참여자 적용 범위를 장기요양 판정 1–5등급으로 넓히고, 의료기관 이용이 어려운 고령층과 지자체 특성에 따라 선별한 사회경제적 취약 노인을 포함하고 있다. 최초 흉부X선에서 의심 소견이 있었으면, 가래검사에서 음성이더라도 6개월 이내 추적검진^2)^ (흉부X선 검사) 실시를 강조하여 환자 조기발견을 강화할 것이며, 노숙인 및 쪽방거주자 대상 결핵환자에 대해서는 임시주거비 지원, 복약관리 등 치료 지원 정책을 지속 운영하여 치료 순응도를 높이고, 치료 중단으로 인한 재발 및 전파를 예방할 계획이다. 이러한 검진–치료–관리 체계로 취약계층 결핵 관리의 효과성을 더욱 높이고자 한다.

찾아가는 결핵검진 사업은 취약계층 내 결핵환자를 조기에 발견하고 치료로 연계하는 데 효과적인 국가-지자체 합동 정책 수단으로 향후 결핵 고위험군 중심의 검진 대상 선정에 근거 자료로 활용할 수 있으며, 추적검진의 확대 및 치료 지원 지속 등을 통해 사업의 효과를 더욱 높일 필요가 있다. 이를 통해 지역사회 전파를 사전에 차단하고, 국가 결핵 발생률 감소 기여도 기대할 수 있을 것이다.

## Figures and Tables

**표 1:** 노인 대상 찾아가는 결핵검진 사업 참여자 및 결핵 확진자의 일반적 특성

구분	검진자		결핵 확진자		
	명(%)		명(%)	발견율	유의수준
전체	167,636 (100.0)		119 (100.0)	[71.0]	
성별					<0.001
남	46,252 (27.6)		55 (46.2)	[118.9]	
여	121,384 (72.4)		64 (53.8)	[52.7]	
연령					<0.001
65–69	9,163 (5.5)		7 (5.9)	[76.4]	
70–74	17,838 (10.6)		3 (2.5)	[16.8]	
75–79	29,341 (17.5)		12 (10.1)	[40.9]	
≥80	111,294 (66.4)		97 (81.5)	[87.2]	
신체활동					0.675
정상	67,962 (40.5)		46 (38.7)	[67.7]	
거동불편	99,674 (59.5)		73 (61.3)	[73.2]	
독거					0.879
해당	81,943 (48.9)		59 (49.6)	[72.0]	
비해당	85,693 (51.1)		60 (50.4)	[70.0]	
기저질환					0.465
있음	122,630 (73.2)		87 (73.1)	[70.9]	
없음	42,954 (25.6)		32 (26.9)	[74.5]	
모름	2,052 (1.2)		0 (0.0)	[0.0]	
결핵 증상(2주 이상 기침)					<0.001
해당	5,351 (3.2)		26 (21.8)	[485.9]	
비해당	162,285 (96.8)		93 (78.2)	[57.3]	
흉부X선 판독 결과					<0.001
정상	125,703 (75.0)		3 (2.5)	[2.4]	
요치료a)/요관찰b)	866 (0.5)		20 (16.8)	[2,309.5]	
비활동성 결핵c)	13,999 (8.4)		88 (73.9)	[628.6]	
기타질환	27,068 (16.1)		8 (6.7)	[29.6]	

단위: 명(%) 혹은 [명/10만 건당]. 반올림으로 인해 백분율의 합이 100%가 되지 않을 수 있음. ^a)^‘활동성 폐결핵’이거나 결핵성으로 추정되는 ‘삼출성 흉막염’으로 나타나 결핵 치료를 권고하는 경우로 확진을 위한 가래검사가 필요함. ^b)^‘활동성미정 폐결핵’ 또는 ‘결핵의심’ 소견이 나타난 경우로 보건소 및 의료기관에서 가래검사를 포함한 추가 결핵검사와 환자의 임상소견 등을 종합한 진료의사의 최종 진단이 필요. 결핵환자로 등록이 안 된 경우에는 1년간 정기적인 추적관찰이 필요함. ^c)^과거에 폐결핵이 발병하였으나, 현재 치유되어 섬유성 병변 등 흔적이 남아 있는 상태임.

**표 2:** 노숙인 및 쪽방거주자 대상 찾아가는 결핵검진 사업 참여자 및 결핵 확진자의 일반적 특성

구분	검진자		결핵 확진자		
	명(%)		명(%)	발견율	유의수준
전체	15,178 (100.0)		18 (100.0)	[118.6]	
성별					0.086
남	11,776 (77.6)		17 (94.4)	[144.4]	
여	3,402 (22.4)		1 (5.6)	[29.4]	
연령					0.827
≤39	530 (3.5)		0 (0.0)	[0.0]	
40–49	1,178 (7.8)		1 (5.6)	[84.9]	
50–59	3,362 (22.2)		6 (33.3)	[178.5]	
60–69	5,158 (34.0)		7 (38.9)	[135.7]	
70–79	3,331 (21.9)		2 (11.1)	[60.0]	
≥80	1,619 (10.7)		2 (11.1)	[123.5]	
검진 대상					0.019
시설노숙인	6,704 (44.2)		3 (16.7)	[44.7]	
거리노숙인	3,817 (25.1)		5 (27.8)	[131.0]	
쪽방거주자	4,657 (30.7)		10 (55.6)	[214.7]	
기저질환					0.273
있음	7,707 (50.8)		6 (33.3)	[77.9]	
없음	7,292 (48.0)		12 (66.7)	[164.6]	
모름	179 (1.2)		0 (0.0)	[0.0]	
결핵 증상(2주 이상 기침)					0.611
해당	215 (1.4)		0 (0.0)	[0.0]	
비해당	14,963 (98.6)		18 (100.0)	[120.3]	
흉부X선 판독 결과					<0.001
정상	12,072 (79.5)		0 (0.0)	[0.0]	
요치료/요관찰	73 (0.5)		3 (16.7)	[4,109.6]	
비활동성 결핵	1,721 (11.3)		15 (83.3)	[871.6]	
기타질환	1,312 (8.6)		0 (0.0)	[0.0]	

단위: 명(%) 혹은 [명/10만 건당]. 반올림으로 인해 백분율의 합이 100%가 되지 않을 수 있음.

**표 3:** 유증상-유소견에 따른 결핵 확진자 발견 현황

흉부X선 판독 결과		결핵 확진자	결핵 증상 유무	
			유	무
전체	총계	137 (100.0)	26 (19.0)	111 (81.0)
	정상	3 (2.2)	3 (100.0)	0 (0.0)
	요치료/요관찰	23 (16.8)	2 (8.7)	21 (91.3)
	비활동성 결핵	103 (75.2)	13 (12.6)	90 (87.4)
	기타질환	8 (5.8)	8 (100.0)	0 (0.0)
노인	소계	119 (100.0)	26 (21.8)	93 (78.2)
	정상	3 (2.5)	3 (100.0)	0 (0.0)
	요치료/요관찰	20 (16.8)	2 (10.0)	18 (90.0)
	비활동성 결핵	88 (73.9)	13 (14.8)	75 (85.2)
	기타질환	8 (6.7)	8 (100.0)	0 (0.0)
노숙인 및 쪽방거주자	소계	18 (100.0)	0 (0.0)	18 (100.0)
	정상	0 (0.0)	0 (0.0)	0 (0.0)
	요치료/요관찰	3 (16.7)	0 (0.0)	3 (100.0)
	비활동성 결핵	15 (83.3)	0 (0.0)	15 (100.0)
	기타질환	0 (0.0)	0 (0.0)	0 (0.0)

단위: 명(%). 반올림으로 인해 백분율의 합이 100%가 되지 않을 수 있음.

## Footnotes

1) 사전문진 작성 시 검진 대상 구분에서 ‘거리노숙인’, ‘쪽방거주자’로 응답한 대상자.

2) 사전문진 작성 시 2주 이상 기침 증상이 있다고 응답한 대상자 또는 흉부X선 판독 결과 결핵 의심 소견(비활동성 결핵 포함)으로 판정을 받은 경우 가래검사를 실시하였으나, 최종 ‘음성’으로 확인된 자.
